# Strawberry and Ginger Silver Nanoparticles as Potential Inhibitors for SARS-CoV-2 Assisted by In Silico Modeling and Metabolic Profiling

**DOI:** 10.3390/antibiotics10070824

**Published:** 2021-07-06

**Authors:** Mohammad M. Al-Sanea, Narek Abelyan, Mohamed A. Abdelgawad, Arafa Musa, Mohammed M. Ghoneim, Tarfah Al-Warhi, Nada Aljaeed, Ohoud J. Alotaibi, Taghreed S. Alnusaire, Sayed F. Abdelwahab, Aya Helmy, Usama Ramadan Abdelmohsen, Khayrya A. Youssif

**Affiliations:** 1 Department of Pharmaceutical Chemistry, College of Pharmacy, Jouf University, Sakaka 72341, Saudi Arabia; mohamedabdelwahab976@yahoo.com; 2 Institute of Biomedicine and Pharmacy, Russian-Armenian University, Yerevan 0051, Armenia; narek.abelyan@rau.am; 3 Foundation for Armenian Science and Technology, Yerevan 0033, Armenia; 4 Department of Pharmacognosy, College of Pharmacy, Jouf University, Sakaka 72341, Saudi Arabia; arafa_1998@yahoo.de; 5 Department of Pharmacognosy, Faculty of Pharmacy, Al-Azhar University, Cairo 11371, Egypt; 6 Department of Pharmacy, College of Pharmacy, Al Maarefa University, Ad Diriyah 13713, Saudi Arabia; mghoneim@mcst.edu.sa; 7 Department of Chemistry, College of Science, Princess Nourah Bint Abdulrahman University, Riyadh 11564, Saudi Arabia; tarfah-w@hotmail.com (T.A.-W.); noaljaeed@pnu.edu.sa (N.A.); ojalotaibi@pnu.edu.sa (O.J.A.); 8 Biology Department, College of Science, Jouf University, Sakaka 72388, Saudi Arabia; tasalnosairi@ju.edu.sa; 9 Olive Research Center, Jouf University, Sakaka 72341, Saudi Arabia; 10 Department of Pharmaceutics and Industrial Pharmacy, Taif College of Pharmacy, Taif University, P.O. Box 11099, Taif 21944, Saudi Arabia; s.fekry@tu.edu.sa; 11 Department of Pharmacognosy, Faculty of Pharmacy, Modern University for Technology and Information, Cairo 11865, Egypt; dr_aya.helmy@hotmail.com (A.H.); khayrya.youssif@gmail.com (K.A.Y.); 12 Department of Pharmacognosy, Faculty of Pharmacy, Deraya University, Minia 61111, Egypt; 13 Department of Pharmacognosy, Faculty of Pharmacy, Minia University, Minia 61519, Egypt

**Keywords:** strawberry, ginger, SARS-CoV-2, nanoparticles, in silico modeling, metabolomic profiling

## Abstract

SARS-CoV-2 (COVID-19), a novel coronavirus causing life-threatening pneumonia, caused a pandemic starting in 2019 and caused unprecedented economic and health crises all over the globe. This requires the rapid discovery of anti-SARS-CoV-2 drug candidates to overcome this life-threatening pandemic. Strawberry (*Fragaria ananassa* Duch.) and ginger (*Zingiber officinale*) methanolic extracts were used for silver nanoparticle (AgNPs) synthesis to explore their SARS-CoV-2 inhibitory potential. Moreover, an in silico study was performed to explore the possible chemical compounds that might be responsible for the anti-SARS-CoV-2 potential. The characterization of the green synthesized AgNPs was carried out with transmission electron microscope (TEM), Fourier-transform infrared, spectroscopy ultraviolet-visible spectroscopy, zeta potential, and a dynamic light-scattering technique. The metabolic profiling of strawberry and ginger methanolic extract was assessed using liquid chromatography coupled with high-resolution mass spectrometry. The antiviral potential against SARS-CoV-2 was evaluated using an MTT assay. Moreover, in silico modeling and the molecular dynamic study were conducted via AutoDock Vina to demonstrate the potential of the dereplicated compounds to bind to some of the SARS-CoV-2 proteins. The TEM analysis of strawberry and ginger AgNPs showed spherical nanoparticles with mean sizes of 5.89 nm and 5.77 nm for strawberry and ginger, respectively. The UV-Visible spectrophotometric analysis showed an absorption peak at λmax of 400 nm for strawberry AgNPs and 405 nm for ginger AgNPs. The Zeta potential values of the AgNPs of the methanolic extract of strawberry was −39.4 mV, while for AgNPs of ginger methanolic extract it was −42.6 mV, which indicates a high stability of the biosynthesized nanoparticles. The strawberry methanolic extract and the green synthesized AgNPs of ginger showed the highest antiviral activity against SARS-CoV-2. Dereplication of the secondary metabolites from the crude methanolic extracts of strawberry and ginger resulted in the annotation of different classes of compounds including phenolic, flavonoids, fatty acids, sesquiterpenes, triterpenes, sterols, and others. The docking study was able to predict the different patterns of interaction between the different compounds of strawberry and ginger with seven SARS-CoV-2 protein targets including five viral proteins (Mpro, ADP ribose phosphatase, NSP14, NSP16, PLpro) and two humans (AAK1, Cathepsin L). The molecular docking and dynamics simulation study showed that neohesperidin demonstrated the potential to bind to both human AAK1 protein and SARS-CoV-2 NSP16 protein, which makes this compound of special interest as a potential dual inhibitor. Overall, the present study provides promise for Anti-SARS-CoV-2 green synthesized AgNPs, which could be developed in the future into a new anti-SARS-CoV-2 drug.

## 1. Introduction

The coronavirus disease 2019 (COVID-19) is caused by the severe acute respiratory syndrome coronavirus-2 (SARS-Cov-2) [1]. At the end of 2019, SARS-CoV-2 caused severe life-threatening pneumonia in Wuhan, China, which spread soon after across the globe in very little time [1]. The SARS-CoV-2 outbreak has seen no signs of receding after spreading to more than 200 countries across the world [2]. The World Health Organization (WHO) announced COVID-19 as a pandemic after three months [2]. Moreover, the Centers for Disease Control and Prevention (CDC) reported that SARS-CoV-2 symptoms range from mild to severe symptoms and death. SARS-CoV-2 symptoms may include a sore throat, fever, shortness of breath, cough, and fatigue [1]. The SARS-CoV-2 mild and moderate cases may represent 81% of total cases, while the severe disease represents about 14% of cases. In about 5% of cases occurs septic shock, respiratory failure, and multiple organ failure [3]. The high mortality rate of SARS-CoV-2 is fundamentally due to the acute respiratory distress syndrome and the severe cytokine release [4,5]. SARS-CoV-2 is a beta coronavirus [6]. Coronaviruses (CoVs) are a family of huge single-stranded, enveloped, and positive-sense viruses (ranging from 27–32 kb), that have distinctive spikes on their surface [7]. Seven strains of human coronaviruses have been identified. Four strains produce mild symptoms: Human coronavirus HKU1(HCoV-HKU1), Human coronavirus OC43 (HCoV-OC43), Human coronavirus NL63 (HCoV-NL63), and Human coronavirus 229E (HCoV-229E), while the other three coronaviruses cause severe symptoms: the Middle East respiratory syndrome (MERS-CoV), severe acute respiratory syndrome coronavirus (SARS-CoV), and severe acute respiratory syndrome coronavirus 2 (SARS-CoV-2) [4]. SARS-CoV-2 uses a densely glycosylated viral protein spike (S) protein to bind to the angiotensin-converting enzyme 2 (ACE2) receptor. Following receptor binding, the virus particle uses host cell receptors and endosomes to enter cells. A host type 2 transmembrane serine protease, TMPRSS2, facilitates cell entry via the S protein [7,8]. The host transmembrane protease serine 2 (TMPRSS2) activates the S protein and assists SARS CoV-2 cell entry [5]. After receptor binding, the virus uses the non-/endosomal pathway to enter the host cells [9]. Once the virus enters the cell, it dissembles intracellularly to release its RNA into the cytoplasm to start the synthesis of the large replicase polyproteins (such as RNA-dependent RNA polymerase (RdRp) and helicase) and for the viral genomic RNA replication [9]. All the viral structural and accessory proteins are synthesized from subgenomic mRNAs. The helical nucleocapsid, genomic RNA, and the other structural proteins compose the assembled virions, which are then released [9]. These viral lifecycle steps (virus entry, synthesis of the large replicase polyproteins, replication of genomic RNA, and assembly of virus) provide remarkable and potential targets for the inhibition of SARS-CoV-2 [10]. Despite so many clinical trials and preclinical studies and all the efforts that have been devoted to developing anti-SARS-Cov-2 agents, up until now there are no approved therapeutics for this infectious disease [9]. The development of COVID-19 vaccines is very important for the world to return to pre-pandemic normalcy, and a mass global effort has been invested into finding a pathway for the protection against SARS-CoV-2. As of March 2021, thirteen vaccines have been approved for application, whilst over 90 vaccine candidates are undergoing clinical trials [10].

Herbal medicines and natural compounds provide a wealthy resource for unprecedented antiviral agents. Some natural medicines possess great antiviral activities against vast virus strains, including herpes simplex virus, coronavirus [11,12,13,14,15,16], influenza virus [17,18], human immunodeficiency virus [19,20,21], hepatitis B and C viruses [22,23,24], SARS and MERS [25,26]. Moreover, numerous studies have reported the antiviral activity of Chinese herbs, as well as hundreds of natural compounds [2]. For example, Emodin is an anthraquinone isolated from *Rheum officinale Baill*. It has antimicrobial and anti-inflammatory effects [27]. Studies have reported that emodin can block S protein binding to ACE2 and reduce the infectivity of S protein pseudo-typed retrovirus to Vero E6 cells with IC_50_ of 200 μM [7]. 

Scutellarein is a flavone isolated from *Scutellaria lateriflora* L. [28] Scientists screened about 64 purified natural compounds that showed inhibitory effects of SARS helicase. Scutellarein inhibited the ATPase activity of nsP13 in vitro, thus potently inhibiting the SARS-CoV helicase protein. However, to validate its efficacy, more preclinical/clinical studies are needed to evaluate its anti-viral activities [7].

Natural herbal medicines have been used for the prevention of virus infection for years. Those herbal medicines show adequate efficacy and tolerable toxicity. It is doubtless the case that herbal medicines are a rich resource for drug discovery, and their tolerable toxicity makes them a potential therapeutic candidate against SARS-CoV-2 [29,30,31,32,33,34,35]. Therefore, this study aimed to provide a new perspective regarding SARS-CoV-2 inhibition using strawberry and ginger green synthesized AgNPs as potential inhibitors against the SARS-CoV-2 pandemic. 

## 2. Material and Methods

### 2.1. Plant Material

#### 2.1.1. Strawberry (*Fragaria ananassa* Duch.)

The leaves of *Fragaria ananassa* Duch. were harvested in April from Moshtohor-Toukh, Al Qalyubiyah, Egypt, and were authenticated by Dr. Mohammed El Gebaly, faculty of Science, Cairo University. Voucher specimen No (00F.A/2020) has been kept in the herbarium of the Pharmacognosy Department, Faculty of Pharmacy, Helwan University.

After harvest, the leaves were washed with tap water, and then surface washed with distilled water to remove any remaining impurities. The leaf parts were shade-dried for 10 days at room temperature to remove the moisture content. Fine powder from the dried leaves was obtained by the use of a clean electric blender and was stored in an airtight, amber glass bottle to avoid sunlight for further use in preparing strawberry methanolic extract.

#### 2.1.2. Ginger (*Zingiber officinale*)

Clean fresh rhizomes of ginger were collected in April from the market, Al Obour, Cairo, Egypt, and authenticated by Dr. Mohammed El Gebaly, faculty of Science, Cairo University. Voucher specimen No (00Z.O/2020) has been kept in the herbarium of the Pharmacognosy Department, Faculty of Pharmacy, Helwan University. The whole mature rhizome was cleaned. The rhizomes of fresh gingers were separately sliced into pieces to prepare the ginger methanolic extract.

However, according to the departmental/institutional/local/national guidelines in Egypt, permissions and approvals are not required for plant research. 

### 2.2. Chemicals

All the reagents used were of an analytical grade and were used without any further purification. Silver nitrate (AgNO_3_) (Purity ≥ 99.5%) was purchased from Sigma-Aldrich, Hamburg, Germany. Methanol, Dimethylsulfoxide (DMSO), and Sodium hydroxide were purchased from Al-Nasr Company for Chemical Industries.

### 2.3. Extraction Procedure

In brief, 100 g of strawberry air-dried leaves and fresh ginger rhizomes were extracted with 80% methanol (3X 2000 mL) at room temperature and the methanolic extracts were concentrated under reduced pressure at 45 °C.

### 2.4. Synthesis of AgNPs 

#### 2.4.1. Synthesis of Strawberry AgNPs Using a Methanolic Extract

Strawberry AgNPs were synthesized by dissolving 0.05 g of strawberry methanolic extract in 3 mL DMSO, then the addition of 0.5 mL of DMSO extract and 1 mL 1 M NaOH to 10 mL 1 mM AgNO_3_. The mixture was kept in a water bath for 10 min at 60 °C.

#### 2.4.2. Synthesis of Ginger AgNPs Using Methanolic Extract

Ginger AgNPs were synthesized by dissolving 0.08 g of strawberry methanolic extract in 3 mL DMSO, and then 1.5 mL of DMSO extract and 1 mL 1 M NaOH to 10 mL 1mM AgNO_3_ was added. The mixture was kept in a water bath for 10 min at 60 °C.

#### 2.4.3. Metabolic Profiling of Strawberry and Ginger Methanolic Extracts

LC-MS profiling was performed on methanolic extracts of strawberry and ginger according to the method described by Haggag et al. [36,37,38,39] on an Acquity Liquid Chromatography system coupled to a Synapt G2 HDMS quadrupole time-of-flight hybrid mass spectrometer (Waters, Milford, CT, USA). The database used for the identification of compounds was the Dictionary of Natural Products.

### 2.5. Antiviral Activity

The antiviral activity was determined by measuring the half-maximal cytotoxic concentration (CC_50_) and Inhibitory Concentration 50 (IC_50_) according to the method described by Feoktistova and Mostafa et al. [29,40].

### 2.6. Characterization of the Synthesized AgNPs by TEM

On a copper grid coated with a carbon support film, a drop of the AgNP solution was added. The size and shape of the AgNPs were analyzed after drying using Transmission Electron Microscope (TEM) Jeol model JEM-1010, the USA at The Regional Center for Mycology and Biotechnology, Al-Azhar University, Cairo, Egypt.

### 2.7. Zeta Potential Characterization of the Synthesized AgNPs of Strawberry and Ginger Methanolic Extracts

The nanoparticles’ surface charge (stability) was studied using a Zeta potential Nano ZS (Malvern instruments) in a disposable cell at 25 °C, and the results were analyzed using Zeta-sizer 7.01 software, at Nawah Scientific, Cairo, Egypt.

### 2.8. Molecular Docking

Crystal structures of AAK1 (PDB ID: 5L4Q), CATHEPSIN L (PDB ID: 5MQY), Mpro (PDB ID: 6LU7), ADP ribose phosphatase (PDB ID: 6W02), NSP 14 (PDB ID: 5C8S), NSP 16 (PDB ID: 6W61), PLpro (PDB ID: 4OW0) proteins were downloaded from the Protein Data Bank. AutoDockTools [11] was used for the generation of the input files of protein structures and co-crystallized ligands and studied compounds. All ligands were processed with OpenBable v2.4 [12]. AutoDock Vina [13,41], which demonstrated high performance in a recent comprehensive assessment of docking programs, was used for molecular docking [18]. Grid boxes whose sizes did not exceed 27,000 Å3 were selected, and “exhaustiveness” was set to 16, which is recommended for rapid virtual screening when using small boxes [19]. Discovery studio was used for visualization and analysis of the interactions of docked compounds to corresponding proteins [42].

### 2.9. Molecular Dynamic Simulations

The molecular dynamic simulations were carried out using AMBER20 [42] with a ff14SB [43] force field for protein and GAFF [44] for the ligand parameterization according to the AM1-BCC [45] scheme order to calculate the atomic point charges. Conformations of selected compounds obtained as the result of molecular docking were used as starting positions for corresponding simulations. As the force field does not contain parameters for the ligand, all ligands were parameterized using an ANTECHAMBER [43,44,45,46] package to generate consistent parameters with the General Amber Force Field [47] (GAFF). The complexes were solvated with TIP3P water and Na+/Cl-ions at 150 mM concentration [48]. The Monte Carlo barostat [49], with reference pressure at 1 bar and a Langevin thermostat [50] with a collision frequency (gamma_ln) 2 ps -1 to keep the temperature at 310.15 K, were used. Particle Mesh Ewald (PME) with electrostatic interactions cut off at 1.0 nm was used for long-range electrostatic interactions. Bonds involving hydrogen were constrained using the SHAKE algorithm and the 2fs integration step was used [51]. Finally, for every simulation, binding free energies were calculated using the Molecular Mechanics/Generalized Born Surface Area (MM/GBSA) method [52] and the MMPBSA.py [53] program, using 250 snapshots with equal intervals collected from the last 25 ns of every trajectory for each simulation. The atom deviations of the ligands were calculated using Root Mean Square Deviations (RMSD) and the relative fluctuations of each amino acid of the proteins were defined with Root Mean Square Fluctuations (RMSF). 

## 3. Results and Discussion

### 3.1. TEM Characterization of the Synthesized AgNPs

**Figure 1:**
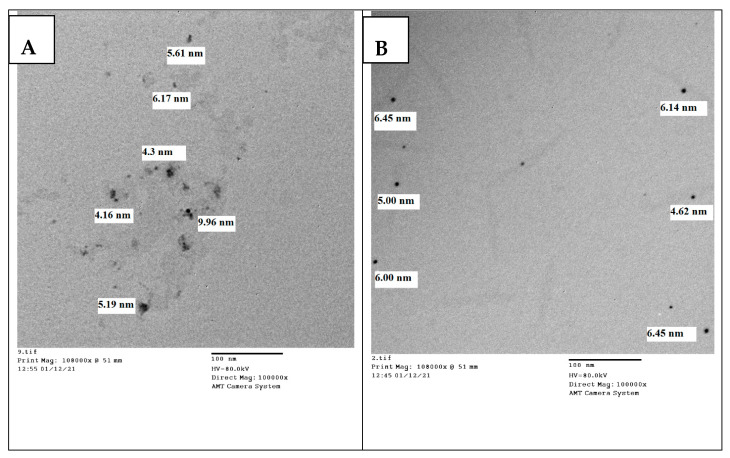
TEM analysis for the shape and size of the synthesized AgNPs of (**A**) methanolic extract AgNPs of strawberry and (**B**) methanolic extract AgNPs of ginger. Abbreviations: TEM, transmission electron microscope; AgNPs, silver nanoparticles.

The TEM analysis of AgNPs of the strawberry and ginger methanolic extracts showed spherical nanoparticles with mean sizes 5.89 nm and 5.77 nm for strawberry and ginger, respectively (Figure 1A,B).

### 3.2. UV-Visible Characterization of the Synthesized AgNPs of Strawberry and Ginger Methanolic Extracts

**Figure 2:**
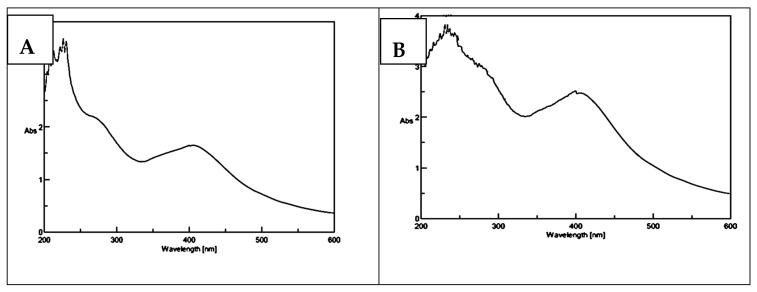
UV-Vis spectral analysis of synthesized (**A**) AgNPs of strawberry and (**B**) AgNPs of ginger methanolic extracts.

The green synthesized AgNP formation was monitored by UV light at a wavelength range from 200–600 nm. The AgNPs of strawberry and ginger methanolic extracts exhibited absorbance bands at 400 nm and 405 nm, respectively. These results proved AgNP synthesis [36] (Figure 2A,B).

### 3.3. Determination of Zeta Potential of Strawberry and Ginger Methanolic Extract AgNPs

**Figure 3:**
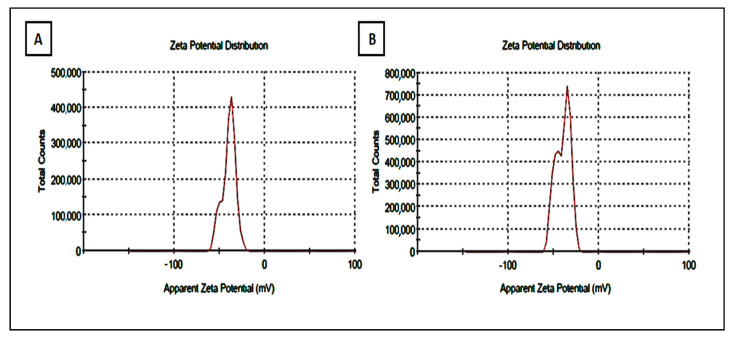
Zeta potential analysis of synthesized SNPs. (**A**) AgNPs of strawberry and (**B**) AgNPs of ginger.

The surface charge of the green synthesized AgNP of strawberry and ginger was determined by measuring the zeta potential (Figure 3A,B). Zeta potential values of the AgNPs of the methanolic extract of strawberry were −39.4 mV, while for AgNPs of ginger methanolic extract it was −42.6 mV [37,43]. These results indicate that the green synthesized AgNPs are negatively charged and completely stable due to the high repulsion power between the nanoparticles [37,44,45,46]. Additionally, when the zeta potential is more than +30 mV and less than −30 Mv, the colloidal solution becomes highly stable [37,47]. 

### 3.4. Metabolomic Profiling of the Crude Methanolic Extracts of Strawberry and Ginger

Dereplication of the secondary metabolites resulted in the annotation of diverse classes of compounds from the crude methanolic extracts of strawberry and ginger. The negative mode has the majority of the identified compounds and revealed the presence of various phenolic, flavonoid, fatty acid, sesquiterpene, triterpene, sterol, and furanocoumarin compounds (Table 1 and Table 2).

### 3.5. Anti-SARS-CoV-2 Activity

To ensure that the used concentration of strawberry and ginger methanolic extracts and the biosynthesized AgNP concentrations are not toxic to the cells, cytotoxicity was assessed on VERO cells using an MTT assay. The CC_50_ for strawberry and ginger methanolic extract is equal to 55.8 μg/mL and 308 μg/mL, respectively. While the CC_50_ for AgNO_3_, Strawberry AgNPs, and ginger AgNPs is equal to 0.000217 μg/mL, 0.202 μg/mL, and 0.252 μg/mL respectively. The anti-SARS-CoV-2 activity was studied using an MTT assay protocol. The strawberry methanolic extract and Ginger AgNPs 0.034 μg/mL showed the highest antiviral activity against SARS-CoV-2 with an IC_50_ of equal value to 0.0062 μg/mL respectively, followed by strawberry AgNPs with an IC_50_ equal to 0.0989 μg/mL. While the methanolic extracts of ginger showed weak antiviral activity against SARS-CoV-2 with an IC_50_ equal to and 206.4 mg/mL respectively.

### 3.6. Molecular Docking Study

A molecular docking study was performed on about 30 compounds against seven SARS-CoV-2 protein targets (Table S1), including five viral proteins (Mpro, ADP ribose phosphatase, NSP14, NSP16, PLpro) and 2 two (AAK1, Cathepsin L). 

Based on the results of the molecular docking of strawberry-identified LC-mass compounds, only in two cases did the docking scores of some of the studied compounds exceed the reference ligand (co-crystallized structures) scores: human AAK1 protein and NSP16 SARS-CoV-2 protein (Table S1). Neohesperidin was the only compound that demonstrated a better docking score compared to the co-crystallized ligand (~N-[5-(4-cyanophenyl)-1~-pyrrolo[2,3-b] pyridin-3-yl]pyridine-3-carboxamide) of the AAK1 protein. In the case of the NSP16 protein, ten compounds showed close or better docking scores than the co-crystallized ligand (s-adenosylmethionine). Three out of these ten compounds showed relatively high similar docking scores: neohesperidin (−9.3 kcal/mol), isovitexin-2″-*O*-rhamnoside (−9.2 kcal/mol), kaempferitrin (−9.2 kcal/mol). 

**Figure 4:**
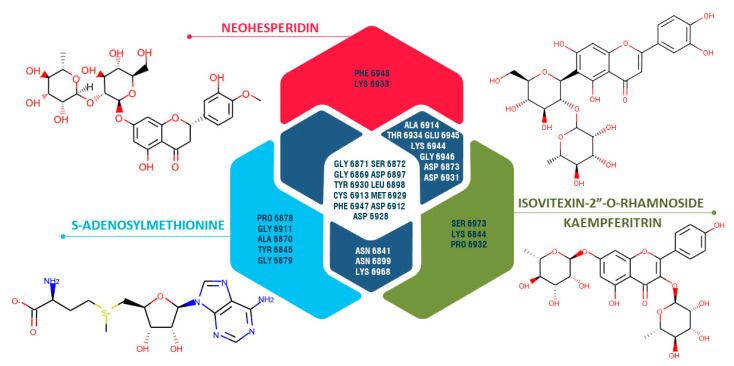
Venn diagram representing unique and common interacting amino acid residues of the SARS CoV-2 NSP 16 (PDB ID: 6W61) binding site with s-adenosylmethionine (co-crystallized ligand), neohesperidin, and isovitexin-2″-*O*-rhamnoside or kaempferitrin based on the results of molecular docking.

Comparative analysis of interacting amino acid residues of NSP 16 binding pocket with studied compounds (Figure 4), showed that PHE 6948, LYS 6933 interact uniquely with neohesperidin, while PRO 6878, GLY 6911, TYR 6845, ALA 6870, and GLY 6879 interact uniquely with co-crystallized ligand (s-adenosylmethionine). 

**Figure 5:**
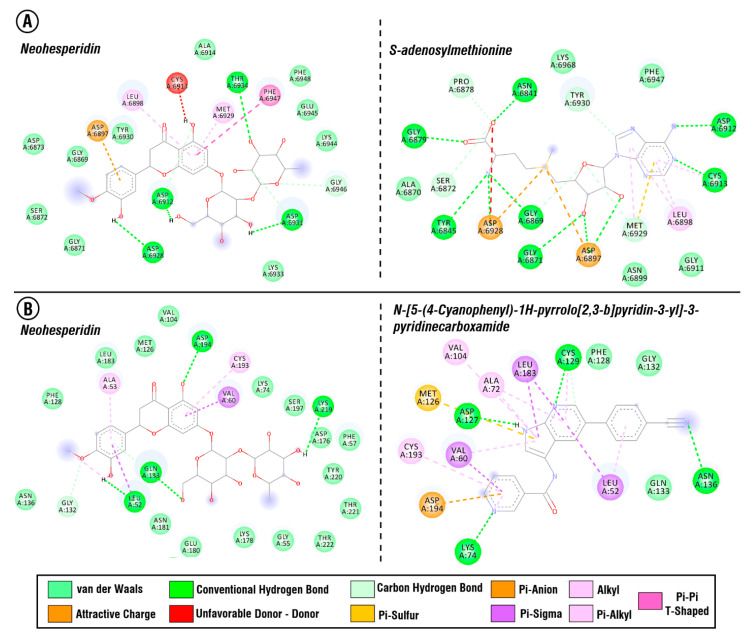
(**A**) Interaction of the neohesperidin and s-adenosylmethionine with the binding site amino acid residues of NSP16 protein, (**B**) Interaction of the neohesperidin and ~N-[5-(4-cyanophenyl)-1~-pyrrolo[2,3-b] pyridin-3-yl] pyridine-3-carboxamide with the binding site amino acid residues of the AAK1 protein.

Neohesperidin has three conventional hydrogen bonds with the following NSP 16 binding site amino acid residues: ASP 6931, THR 6934, ASP 6928 (Figure 5). In comparison, co-crystallized ligand (s-adenosylmethinonine) has six conventional hydrogen bonds with the following amino acid residues: ASN 6841, GLY 6879, TYR 6845, GLY 6869, GLY6871, CYS 6913. Besides, both of these compounds have a conventional hydrogen bond with ASP 6912. Nonetheless, neohesperidin showed a better docking score, which is probably driven by more favorable electrostatic interactions. Isovitexin-2″-*O*-rhamnoside has two conventional hydrogen bonds with THR 6934 and kaempferitrin has three hydrogen bonds with ASP 6912 and CYS 6913 of the NSP 16 binding site (S. 2&3). Electrostatic interactions also play a noticeable role in the binding of these two compounds.

Neohesperidin and ~N-[5-(4-cyanophenyl)-1~{H}-pyrrolo[2,3-b] pyridin-3-yl] pyridine-3-carboxamide (reference ligand) demonstrated close docking score binding energies to AAK1 protein. Each of the compounds have four conventional hydrogen bonds with amino acid residues of the AAK1 protein binding site. Neohesperidin has hydrogen bonds with ASP 194, LYS 219, GLN 133, LEU 52, while co-crystallized ligand of AAK1 protein binds to LYS 74, ASN 136, CYS 129, ASP 127. 

Based on the results of molecular docking, some of the ginger LC-mass identified compounds have demonstrated a potential to bind to the studied proteins. Quercetin showed a slightly higher score compared to the co-crystallized ligand of NSP16 (S-Adenosylmethionine), riboflavin and 5,6-epoxy cholestan-3-ol demonstrated relatively close docking scores. Quercetin also showed a close docking score to the co-crystallized ligand of AAK1. As a result of molecular docking quercetin, 5,6-epoxy cholestan-3-ol, riboflavin, 1,7-bis-(4-Hydroxy-3-methoxyphenyl)-hepta-1,6-diene-3,5-dione, gingerenone-A almost in all cases were placed within the top 5 compounds for all studied proteins. 

**Figure 6:**
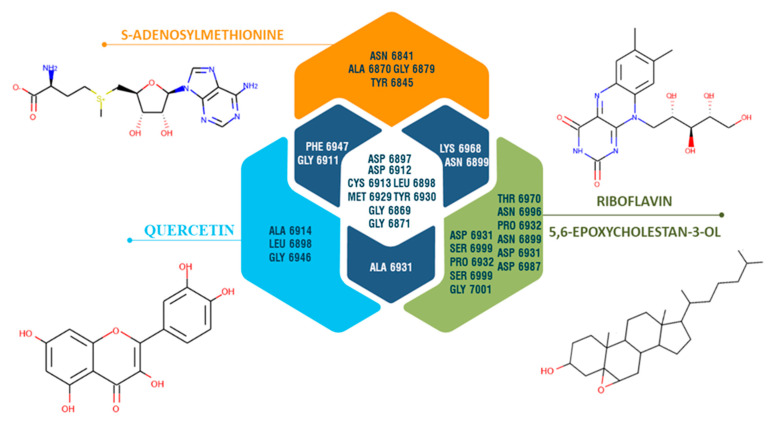
Venn diagram representing unique and common interacting amino acid residues of the SARS-CoV-2 NSP 16 (PDB ID: 6W61) binding site with s-adenosylmethionine (co-crystallized ligand), quercetin, riboflavin, and 5,6-epoxycholestan-3-ol) based on the results of molecular docking.

Comparative analysis of interacting amino acid residues of the NSP 16 binding site with studied compounds (Figure 6) showed that ALA 6914, LEU 6898, and GLY 6946 interact uniquely with quercetin, while ASN 6841, ALA 6870, GLY 6879, and TYR 6845 interact with co-crystallized ligands (s-adenosylmethionine). PHE 6947 and GLY 6911 showed an interaction with quercetin and the co-crystallized ligand and did not show an interaction with other studied compounds. ASP 6912 and 6897, GLY 6869 and 6871, and also LEU 6898, TYR 6930, CYS 6913, and MET 6929 are common interacting amino acid residues for co-crystallized ligands, quercetin, and other compounds (riboflavin and 5,6-epoxycholestan-3-ol).

**Figure 7:**
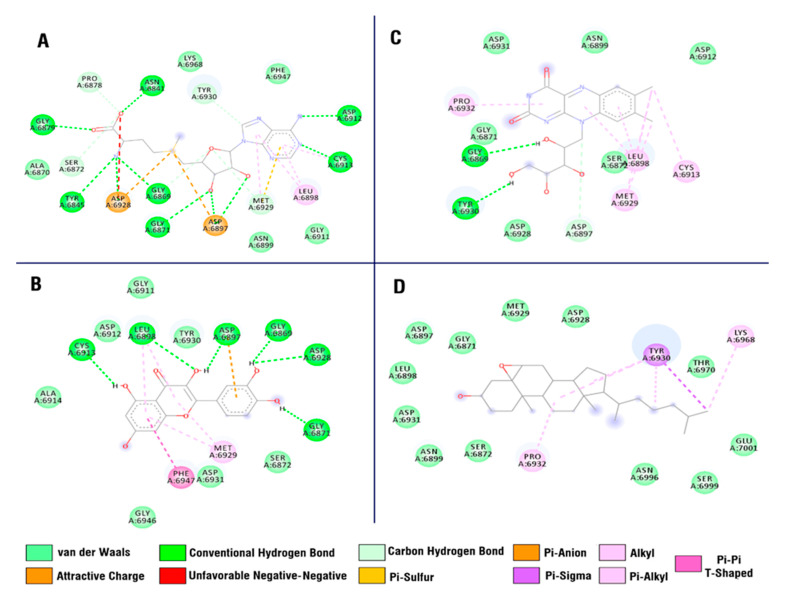
Interaction of (**A**) S-Adenosylmethionine, (**B**) Quercetin, (**C**) Riboflavin, (**D**) 5,6-epoxy cholestan-3-ol with SARS-CoV-2 NSP 16 protein.

Detailed analysis of the interaction of the NSP 16 binding site amino acid residues with top compounds is presented in (Figure 7). In comparison to s-adenosylmethionine (co-crystallized ligand), quercetin has a hydrogen bond with LEU 6898 (Figure 7B), while s-adenosylmethionine, additional to common interacting amino residues between two molecules, forms hydrogen bonds with ASN 6841, GLY 6879, and TYR 6845 (Figure 7A).

Furthermore, some of the top scoring compounds, such as neohesperidin, kaempferitrin and epicatechin 5-O-β-D-glucopyranoside-3-benzoate, are likely not chemically stable in alkaline (NaOH) solutions and can undergo chemical modifications. Kaempferitrin (kaempferol-3,7-dirhamnoside) and neohesperidin are both 7-O-glycosides and can thus undergo hydrolysis at the 7-O-glycoside bond. Epicatechin 5-O-β-D-glucopyranoside-3-benzoate as an ester can hydrolyze into the Epicatechin 5-O-β-D-glucopyranoside and benzoate in the presence of NaOH at 60 °C. All of these compounds were also docked against the investigated proteins. 

As a result of molecular docking, the study neohesperidin demonstrated the potential to bind to both human AAK1 protein and SARS-CoV-2 NSP16 protein, which makes this compound of special interest as a potential dual inhibitor. 

Physicochemical properties and drug-likeness of studied compounds were tested using SwissADME [43].

### 3.7. Molecular Simulation Study

**Figure 8:**
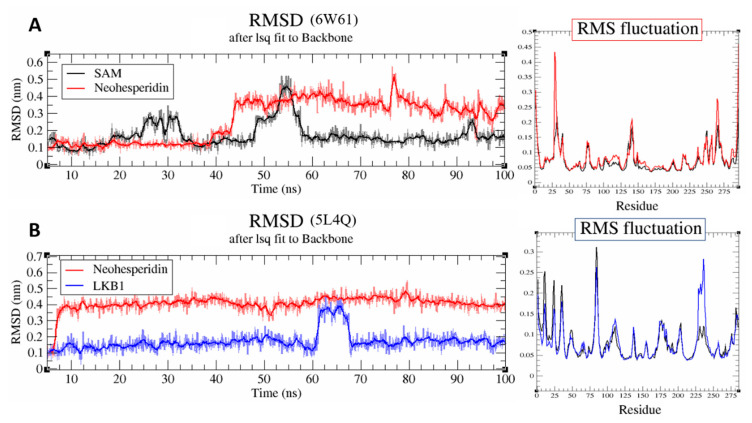
RMSD and RMSF plots of neohesperidin and reference ligands during 100 ns of molecular dynamic simulations in complex with SARS CoV-2 NSP 16 (PDB ID: 6W61) and human Adaptor Protein 2 Associated Kinase 1 (PDB ID: 5L4Q).

Neohesperidin demonstrated a stable interaction with both SARS CoV-2 NSP 16 and human AAK1 based on the average deviation of the RMSD values (<0.2 nm) during the 100 ns simulations (Figure 8). In both cases, neohesperidin undergoes conformational changes in the case of NSP 16 protein at ~45 ns (Figure 8A), and in the case of AAK1 protein at ~5 ns (Figure 8B) of the performed simulation.

**Figure 9:**
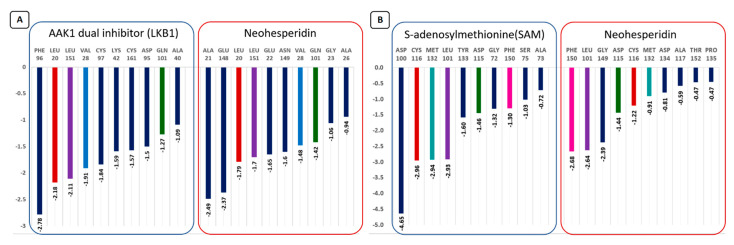
Binding energies (kcal/mol) of the interacting amino acid residues (10 residues with the highest binding energies) of the AAK1 protein’s ligand-binding site with neohesperidin and reference ligand (LKB1).

Besides, to identify the flexible and rigid regions of the complex, RMSF analysis was performed to measure the average atomic flexibility of the Cα atoms of the docked complexes (Figure 9). Both complexes demonstrated a similar average deviation of RMSF values (<0.2 nm). The overall results of RTSD and RSMF showed that the neohesperidin reaches a stable dynamic equilibrium within 100 ns of the performed simulations. 

For the detailed analysis of interactions between Neohesperidin with SARS CoV-2 NSP16 and human AAK1 proteins, binding energies were calculated using an MM/GBSA method (Table 3). Comparative analysis showed that in the case of the human AAK1 protein, neohesperidin interacts in a similar way to the reference total binding energy (−41.43 and −42. 7 kcal/mol respectively, Table 3). With NSP16 viral protein, neohesperidin interacts with a total binding energy of −31.51 kcal/mol, while the reference ligand (SAM) interacts with a higher total binding energy (−51.94 kcal/mol). Detailed information on the interaction types between studied compounds and proteins and corresponding values are presented in Table 1.

To distinguish the key amino acid residues that play an essential role in binding, the energetic contributions of all amino acid residues to the ligand-binding were calculated (Figure 9).

To carry out a comparative analysis of the interaction of neohesperidin and the co-crystallized ligand (LKB1) with the AAK1 protein, 10 amino acid residues (with a relatively high binding energy) were selected. The same four amino acid residues (LEU 20, LEU 151, VAL 28, GLN 101) had similar binding energies for both compounds. PHE 96 interacts with a co-crystallized ligand with a high negative binding energy of ~−2.78 kcal/mol. ALA 21 and GLU 148 showed relatively high binding energy values with neohesperidin with energies of −2.49 and −2.37 kcal/mol, respectively (Figure 9).

In the case of the SARS CoV-2 NSP16 protein’s ligand-binding site with neohesperidin and reference ligand (SAM), the same five amino acid residues, CYS 116, MET 132, LEU 101, ASP 115, and PHE 150, interact with both compounds (Figure 9). Neohesperidin interacts with the PHE 150 with a relatively higher binding energy than the reference ligand. 

As a result of the molecular docking and dynamic simulations study, neohesperidin demonstrated the potential to bind to both the human AAK1 protein and SARS-CoV-2 NSP16 protein, which makes this compound of special interest as a potential dual inhibitor.

## 4. Discussion

Silver is a unique chemical element that has several applications in numerous fields such as electronics, medicine, and household applications. For example, silver sulfadiazine has been used for the treatment of burn wounds to act as an antibiofilm on the wound area; therefore, it enhances the wound healing process [44]. In the last few years, there has been a marvelous rise in the application of nanoscience and nanotechnologies. This rise had led to using silver on the nanoscale and this has produced nanoparticles that have attracted huge interest due to their unparalleled chemical, physical, and biological properties in comparison with their macro-scaled counterparts [65]. Evaluation and characterization of the green-synthesized silver nanoparticles (AgNPs) includes various analytical techniques, including transmission electron microscopy (TEM), which shows spherical AgNPS and ultraviolet-visible spectroscopy (UV–Vis spectroscopy) at the range of 400–500 nm, zeta potential when the value is more than +30 mV and less than −30 mV and Fourier transform infrared spectroscopy (FTIR) in the wavelength range of 400–4000 cm^−1^, which shows the functional groups attached to the surface of AgNPs [45]. All these analytical techniques are crucial to evaluate the bio-distribution, behavior, and reactivity of the synthesized nanoparticles [45]. The morphology, size, and distribution of nanoparticles affect the physicochemical properties of AgNPs, which can be controlled through using different synthesis methods, reducing agents, and stabilizers [46]. Additionally, the nanoparticle size is an important factor of biological activity. For example, it is preferred that the size be greater than 100 nm to conciliate for the drug quantity to be delivered [49]. Moreover, AgNPs are size-dependent and they show a higher toxicity when they get smaller in size due to the high ion release inside the living cells [54]. Moreover, AgNPs have antimicrobial applications and can be used in the fields of nanomedicine, pharmacy, and healthcare [47]. Recently, the outbreaks of new infectious diseases have encouraged numerous researches into the development of new antiviral therapeutics. Viral infection occurs when the viral surface components bind with ligands and proteins on the cell membrane. Therefore, the best strategy for developing antiviral drugs is to inhibit the interaction between the virus ligand and the cell membrane, thereby preventing the virus entry into the cell [45]. Silver nanoparticles are one of the strongest candidates used as antiviral agents. Natural products, especially plant secondary metabolites, have been extensively studied for their efficacy to synthesize the silver nanoparticle (AgNPs). Secondary metabolites are mainly found in plants, specifically plant flavonoids, which have a significant role in the synthesis of AgNPs. The flavonoid-based AgNPs have gained much attention in the last few years [55].

Flavonoids are categories of plant active constituents that are produced to nullify the stress conditions. The polyhydroxyl group of these secondary metabolites is responsible for their free radical scavenging and antioxidant activities, which act as a major contributor in reducing metal ions into NPs [56]. Different researchers have reported that functional groups of flavonoids with a low molecular weight aliphatic and aromatic characteristics are the key contributors in the AgNPs synthesis [74,75]. Flavonoid-based silver nanoparticles (FlavAgNPs) have crucial implications in the green synthesis of AgNPs. Due to the absence of authentic documentation of accurate mechanism, it is supposed that flavonoids act in the capacity of surface-active molecules to reduce and stabilize the AgNPs [55]. However, it is well known that Flavonoids undergo a chemical modification in an alkaline aqueous solution, consisting of an autoxidation that mainly affects the C-ring. This process occurs at a pH above 11.0 and implies the formation of a benzofuranone as a first step, which can further undergo fragmentation, leading to simpler molecular products, which can then undergo a subsequent polymerization. The Raman spectral analysis of different flavonoids showed that several important structural points determine the reactivity of the flavonoid molecules at an alkaline pH: (i) the C3–OH group in the C-ring; (ii) the catechol moiety in the B-ring; and (iii) the C2–C3 bond in the C-ring. For example, quercetin possesses all these groups and exhibits a high instability in alkaline solutions. The reactivity of the C3–OH group is enhanced by the presence of a catechol moiety in the B-ring. If the molecule possesses the catechol group but lacks the C3–OH group, the chemical modifications are minor and they are dominated by a deprotonation of the molecule. The lack of the C2–C3 double bond gives rise to different chemical modifications. Additionally, the SERS spectra of quercetin recorded on AgNPs reveal that this molecule undergoes a large chemical modification on metal AgNPs, even at a neutral pH, mainly involving the dimerization and possible oligomerization of the flavonoid on the surface where the A- and C-rings have a high importance. At an alkaline pH, the spectral changes revealed a change in the dimerization protocol of quercetin, with a possible key role for the B-ring. It is difficult in these cases to discern the effect of deprotonation from that of polymerization [57].

Moreover, there are a few numbers of researches on the effects of AgNPs against various types of viruses. However, the details of the interactions are so limited [45]. Silver nanoparticles may interact with infective viruses in two ways: they may bind to the viral outer coat and prohibit the attachment of the virus towards the cell receptors, or silver nanoparticles bind to the viral DNA or RNA, thus inhibiting the viral replication and propagation inside the living cell [45]. The strong antiviral activities of AgNPs could lower the risk of infection or might also prevent a pandemic viral disease, such as SARS-CoV-2. Sarkar et al. [68] have already suggested the use of AgNPS as a therapeutic agent for the treatment of SARS-CoV-2 infection [50]. The theory was that the AgNPs will bind to the viral spike glycoprotein and thus inhibits the binding of the virus into the living cells, and consequently, the release of silver ions will decrease the pH of respiratory epithelium, where the COVID-19 virus usually lives, to become more acidic and therefore antagonistic towards the virus [50]. In the same way, our results also demonstrated the marked antiviral potential of strawberry and ginger AgNPs with IC_50_ 0.0989 μg/mL, and 0.034 μg/mL, respectively. The high efficacy of the green synthesized AgNPs gives a new window for finding new therapeutic drugs that can contribute to controlling the SARS-CoV-2 pandemic. 

Additionally, studies proved that one of the distinctive features of the eukaryotic mRNAs is characterized by the presence of a methylated 50-cap structure that is required for mRNA stability. Viruses that infect eukaryotic organisms generally modify the 50-cap of viral RNAs to mimic the host’s mRNA structure. In this manner, the virus protects itself from degradation by 50–30 exoribonucleases, enabling efficient translation, and escapes recognition by the host immune system. SARS-CoV-2 encodes one SAM-dependent methyltransferase (a 20O-MTase also known as NSP16) that methylates the RNA cap at ribose 20O positions. Importantly, this enzyme is only active in the presence of its activating partner, the non-structural protein NSP10. The formation of the NSP16/NSP10 complex, which follows a 1:1 stoichiometry ratio, has only been identified in coronaviruses [58]. Therefore, developing small molecule inhibitors of Nsp16 is a promising therapeutic strategy. In the present study, experiments have shown that neohesperidin has three conventional hydrogen bonds with the NSP 16 binding site amino acid residues: ASP 6931, THR 6934, and ASP 6928. Besides, it binds to ASP 6912 with a conventional hydrogen bond. These results suggest neohesperidin as a potential inhibitor for NSP16.

Finally, the Adaptor-Associated Kinase 1 (AAK1). AAK1 is one of the host serine−threonine protein kinases that regulate intracellular viral trafficking during the entry, assembly, and release of multiple unrelated RNA viruses. AAK1 plays a key role in receptor-mediated endocytosis by specific phosphorylation of adaptor protein 2, which stimulates the binding to cargo proteins [57]. Recently, AAK1 inhibition has been proposed for the treatment of dengue and Ebola patients in future outbreaks. However, these have not been included in the present clinical trials. Baricitinib, a potent AAK1 inhibitor, has been proposed as an effective therapy for COVID-19, reducing the viral entry, although no experimental work has been done to prove its mechanism of action [57]. During this work, molecular docking studies have shown that neohesperidin can bind to AAk1 amino acid residues ASP 194, LYS 219, GLN 133, and LEU through four conventional hydrogen bonds. In conclusion, we can find from the previous discussion that neohespiridin may act as a dual inhibitor for both the viral NSP16 and AAK1 and can be a potential therapeutic drug against the SARS-CoV-2 pandemic. 

## 5. Conclusions 

The strawberry (*Fragaria ananassa* Duch.) and ginger (*Zingiber officinale*) silver nanoparticles exhibited an inhibitory potential against SARS-CoV-2. The molecular docking and dynamic simulation study reported that neohesperidin demonstrated the potential to bind to both human AAK1 protein and SARS-CoV-2 NSP16 protein, which makes this compound a promising candidate for future clinical work. 

## Figures and Tables

**Table 1:** Compounds annotated from the methanolic extract of strawberry.

M/Z	Retention Time (min.)	M.wt.	Name	Molecular Formula	References
557.27576	1.7060625	556.26849	Epicatechin 5-*O*-beta-D-glucopyranoside-3-benzoate	C_28_H_28_O_12_	[38]
611.26602	1.8878542	610.25874	Neohesperidin	C_28_H_34_O_15_	[54]
579.27182	2.1045125	578.26455	Kaempferol-3,7-dirhamnoside (Kaempferitrin)	C_27_H_30_O_14_	[37]
595.26792	2.1045083	594.26064	Quercetin-3-O-neohesperidoside	C_27_H_30_O_16_	[55]
283.23742	2.1895583	282.23015	Oleic acid	C_18_H_34_O_2_	[56]
185.12046	2.2322833	184.11318	3,4,5-Trimethoxyphenol	C_9_HO_4_	[57]
291.14876	2.4932	290.14149	Epicatechin	C_15_H_14_O_6_	[58]
305.13074	2.6144542	304.12347	Oxypeucedanin hydrate	C_16_H_16_O_6_	[59]
369.10693	2.6189167	368.09965	3-Feruloylquinic acid	C_17_H_20_O_9_	[60]
289.13343	2.8026875	288.12616	Eriodictyol	C_15_H_12_O_6_	[61]
271.11876	2.8048708	270.11148	Apigenin	C_15_H_10_O_5_	[62]
287.11667	3.1027417	286.1094	Luteolin	C_15_H_10_O_6_	[63]
295.166	4.4977708	294.15873	C-Methyl flavone	C_19_H_18_O_3_	[64]
427.44564	6.6987333	426.43837	β- amyrin	C_30_H_50_O	[65]
579.2612	8.2327222	578.25392	Isovitexin-2″-*O*-rhamnoside	C_27_H_30_O_14_	[48]

**Table 2:** Compounds annotated from the methanolic extract of ginger.

M/Z.	Retention Time (min.)	M.wt.	Name	Molecular Formula	References
195.10038	3.470625	194.0931	Zingerone	C_11_H_14_O_3_	[49]
357.13919	5.799025	356.13191	Gingerenone-A	C_21_H_24_O_5_	[50]
267.15755	6.4666167	266.15027	4-Gingerol	C_15_H_22_O_4_	[51]
293.17373	6.8985917	292.16646	6-Gingerdione	C_17_H_24_O_4_	[52]
347.23134	7.1113167	346.22406	1-Dehydro-(10) gingerdione	C_21_H_30_O_4_	[53]
219.13866	8.0879333	218.13138	Zerumbone	C_15_H_22_O	[66]
291.19394	8.3226583	290.18666	6-Dehydrogingerdione	C_17_H_22_O_4_	[52]
333.25208	8.4842667	332.2448	10-Shogaol	C_21_H_32_O_3_	[52]
237.18401	8.8010667	236.17673	Spiro [4.5] decan-7-one, 1,8- Dimethyl-8,9-epoxy-4-isopropyl	C_15_H_24_O_2_	[67]
369.22559	9.3268667	368.21831	1,7-Bis-(4-Hydroxy-3-methoxyphenyl)-hepta-1,6-diene-3,5-dione	C_21_H_20_O_6_	[68]
219.17552	9.5892	218.16825	Nuciferol	C_15_H_22_O	[69]
293.2097	9.767	292.20242	7-Paradol	C_18_H_28_O_3_	[70]
303.14746	9.8031667	302.14018	Quercetin	C_15_H_10_O_7_	[71]
403.34227	12.798483	402.33499	Cholestan-3-ol, 5,6-epoxy-, (3.beta.,5.alpha.,6.alpha.)	C_27_H_46_O_2_	[72]
377.17929	16.717167	376.17201	Riboflavin	C_17_H_20_N_4_O_6_	[73]

**Table 3:** Different interaction energy types and total binding energies of neohesperidin and reference ligands of SARS CoV-2 NSP16 and human AAK1 proteins.

Protein	Ligand	VDWAALS (kcal/mol)	EEL (kcal/mol)	EGB (kcal/mol)	ESURF (kcal/mol)	ΔG Gas (kcal/mol)	ΔG Solv (kcal/mol)	ΔTOTAL (kcal/mol)
AAK1	LKB1 (reference)	−46.4	−27.03	36.61	−5.89	−73.43	30.72	−42.7
	Neohesperidin	−52.33	−31.55	49.01	−6.56	−83.88	42.45	−41.43
NSP16	SAM (reference)	−46.3	−88.43	88.72	−5.93	−134.73	82.8	−51.94
	Neohesperidin	−33.74	−48.01	55.07	−4.83	−81.75	50.24	−31.51

## Supplementary Materials

The following are available online at https://www.mdpi.com/article/10.3390/antibiotics10070824/s1, Table S1: The scores of re-docking the co-crystallized ligands and the top-scoring compounds of the strawberry and ginger methanolic extracts against seven SARS-CoV-2 protein targets.
