# Supplementary material for: Strawberry and Ginger Silver Nanoparticles as Potential Inhibitors for SARS-CoV-2 Assisted by In Silico Modeling and Metabolic Profiling
Source: Antibiotics (Basel). 2021 Jul 6;10(7):824. doi: 10.3390/antibiotics10070824 (PMC8300822; doi:10.3390/antibiotics10070824)
Supplement: Supplementary file 1 [file antibiotics-10-00824-s001.zip › antibiotics-1199201-supplementary.pdf]

antibiotics

MDPI

Supplementary

Strawberry and Ginger Silver Nanoparticles as Potential Inhibitors for SARS-CoV-2 Assisted by in Silico Modeling and Metabolic Profiling

Mohammad M. Al-Sanea [1,*], Narek Abelyan [2,3], Mohamed A. Abdelgawad [1], Arafa Musa [4,5], Mohammed M. Ghoneim [6], Tarfah Al-Warhi [7], Nada Aljaeed [7], Ohoud J. Alotaibi [7], Taghreed S. Alnusaire [8,9], Sayed F. Abdelwahab [10], Aya Helmy [11], Usama Ramadan Abdelmohsen [12,13,*] and Khayrya A. Youssif [11]

1 Department of Pharmaceutical Chemistry, College of Pharmacy, Jouf University, Sakaka 72341, Saudi Arabia; mohamedabdelwahab976@yahoo.com
2 Institute of Biomedicine and Pharmacy, Russian-Armenian University, Yerevan 0051, Armenia; narek.abelyan@rau.am
3 Foundation for Armenian Science and Technology, Yerevan 0033, Armenia
4 Department of Pharmacognosy, College of Pharmacy, Jouf University, Sakaka 72341, Saudi Arabia; arafa_1998@yahoo.de
5 Department of Pharmacognosy, Faculty of Pharmacy, Al-Azhar University, Cairo 11371, Egypt
6 Department of Pharmacy, College of Pharmacy, Al Maarefa University, Ad Diriyah 13713, Saudi Arabia; mghoneim@mcst.edu.sa
7 Department of Chemistry, College of Science, Princess Nourah Bint Abdulrahman University, Riyadh 11564, Saudi Arabia; tarfah-w@hotmail.com (T.A.-W.); noaljaeed@pnu.edu.sa (N.A.); ojalotaibi@pnu.edu.sa (O.J.A.)
8 Biology Department, College of Science, Jouf University, Sakaka 72388, Saudi Arabia; tasalnosairi@ju.edu.sa
9 Olive Research Center, Jouf University, Sakaka 72341, Saudi Arabia
10 Department of Pharmaceutics and Industrial Pharmacy, Taif College of Pharmacy, Taif University, P.O. Box 11099, Taif 21944, Saudi Arabia; s.fekry@tu.edu.sa
11 Department of Pharmacognosy, Faculty of Pharmacy, Modern University for Technology and Information, Cairo 11865, Egypt; dr_aya.helmy@hotmail.com (A.H.); khayrya.youssif@gmail.com (K.A.Y.)
12 Department of Pharmacognosy, Faculty of Pharmacy, Deraya University, Minia 61111, Egypt
13 Department of Pharmacognosy, Faculty of Pharmacy, Minia University, Minia 61519, Egypt
* Correspondence: mmalsanea@ju.edu.sa (M.M.A.-S.); usama.ramadan@mu.edu.eg (U.R.A.)

Citation: Al-Sanea, M.M.; Abelyan, N.; Abdelgawad, M.A.; Musa, A.; Ghoneim, M.M.; Al-Warhi, T.; Aljaeed, N.; Alotaibi, O.J.; Alnusaire, T.S.; Abdelwahab, S.F.; et al. Strawberry and Ginger Silver Nanoparticles as Potential Inhibitors for SARS-CoV-2 Assisted by In Silico Modeling and Metabolomic Profiling. *Antibiotics* **2021**, *9*, 824. https://doi.org/10.3390/antibiotics10070824

Academic Editor: Mohamed-Elamir F. Hegazy

Received: 11 April 2021
Accepted: 29 June 2021
Published: 6 July 2021

Publisher's Note: MDPI stays neutral with regard to jurisdictional claims in published maps and institutional affiliations.

Copyright: © 2021 by the authors. Submitted for possible open access publication under the terms and conditions of the Creative Commons Attribution (CC BY) license (http://creativecommons.org/licenses/by/4.0/).

Abstract: SARS-CoV-2 (COVID-19), a novel coronavirus causing life-threatening pneumonia, caused a pandemic starting in 2019 and caused unprecedented economic and health crises all over the globe. This requires the rapid discovery of anti-SARS-CoV-2 drug candidates to overcome this life-threatening pandemic. Strawberry (*Fragaria ananassa* Duch.) and ginger (*Zingiber officinale*) methanolic extracts were used for silver nanoparticle (AgNPs) synthesis to explore their SARS-CoV-2 inhibitory potential. Moreover, an in silico study was performed to explore the possible chemical compounds that might be responsible for the anti-SARS-CoV-2 potential. The characterization of the green synthesized AgNPs was carried out with transmission electron microscope (TEM), Fourier-transform infrared, spectroscopy ultraviolet-visible spectroscopy, zeta potential, and a dynamic light-scattering technique. The metabolic profiling of strawberry and ginger methanolic extract was assessed using liquid chromatography coupled with high-resolution mass spectrometry. The antiviral potential against SARS-CoV-2 was evaluated using an MTT assay. Moreover, in silico modeling and the molecular dynamic study were conducted via AutoDock Vina to demonstrate the potential of the dereplicated compounds to bind to some of the SARS-CoV-2 proteins. The TEM analysis of strawberry and ginger AgNPs showed spherical nanoparticles with mean sizes of 5.89 nm and 5.77 nm for strawberry and ginger, respectively. The UV-Visible spectrophotometric analysis showed an absorption peak at λmax of 400 nm for strawberry AgNPs and 405 nm for ginger AgNPs. The Zeta potential values of the AgNPs of the methanolic extract of strawberry was −39.4 mV, while for AgNPs of ginger methanolic extract it was −42.6 mV, which indicates a high stability of the biosynthesized nanoparticles. The strawberry methanolic extract and the green synthesized AgNPs of ginger showed the highest antiviral activity against SARS-CoV-2. Dereplication of the secondary

metabolites from the crude methanolic extracts of strawberry and ginger resulted in the annotation of different classes of compounds including phenolic, flavonoids, fatty acids, sesquiterpenes, triterpenes, sterols, and others. The docking study was able to predict the different patterns of interaction between the different compounds of strawberry and ginger with seven SARS-CoV-2 protein targets including five viral proteins (Mpro, ADP ribose phosphatase, NSP14, NSP16, PLpro) and two humans (AAK1, Cathepsin L). The molecular docking and dynamics simulation study showed that neohesperidin demonstrated the potential to bind to both human AAK1 protein and SARS-CoV-2 NSP16 protein, which makes this compound of special interest as a potential dual inhibitor. Overall, the present study provides promise for Anti-SARS-CoV-2 green synthesized AgNPs, which could be developed in the future into a new anti-SARS-CoV-2 drug.

Keywords: strawberry; ginger; SARS-CoV-2; nanoparticles; in silico modeling; metabolomic profiling

1. Methods:

1.1. Metabolomic Profiling of Strawberry and Ginger Methanolic Extracts

Chromatographic separation was carried out on a BEH C18 column (2.1 × 100 mm, 1.7 μm particle size; Waters, Milford, USA) with a guard column (2.1 × 5 mm, 1.7 μm particle size) and a linear binary solvent gradient of 0–100% eluent B over 6 mins at a flow rate of 0.3 mL min−1, using 0.1% formic acid in water (v/v) as solvent A and acetonitrile as solvent B. The injection volume was 2 μL and the column temperature was 40 °C. To convert the raw data into separate positive and negative ionization files, MSConvert software was used. The files were then imported to the data mining software MZmine 2.10 for peak picking, deconvolution, deisotoping, alignment, and formula prediction.

2. Antiviral Activity

2.1. Virus and Cells.

Vero-E6 cells were maintained in Dulbecco's Modified Eagle's medium (DMEM) containing 10% Fetal Bovine Serum (FBS) (Invitrogen) and 2% Penicillin/Streptomycin (pen/strep) antibiotic mixture at 37 °C, 5% CO_2. For preparing virus stock, cells were distributed into tissue culture flasks 24 h before infection with hCoV-19/Egypt/NRC-3/2020 isolate at a multiplicity of infection (MOI) of 0.1 in infection medium (DMEM containing 4% FBS, 1% pen/strep, and 1% L-1-tosylamide-2-phenylethyl chloromethyl ketone (TPCK)-treated trypsin). Two hours later, the infection medium containing virus inoculum was removed and replaced with a fresh infection medium and incubated for three days. At the indicated time point, cell supernatant was collected and centrifuged for 5 min at 2500 rpm to remove small particulate cell debris. The supernatant was transferred to a fresh 50 ml falcon tube, aliquoted, and titrated using plaque infectivity assay.

2.2. MTT Cytotoxicity Assay

To assess the half-maximal cytotoxic concentration (CC_{50}), stock solutions of the test compounds were prepared in 10% DMSO in ddH_2O and diluted further to the working solutions with DMEM. The cytotoxic activity of the extracts was tested in Vero-E6 cells by using the 3-(4, 5-dimethylthiazol-2-yl)-2, 5-diphenyltetrazolium bromide (MTT) method with minor modifications. Briefly, the cells were seeded in 96-well plates (100 μL/well at a density of 3 × 105 cells/ml) and incubated for 24 h at 37 °C in 5% CO_2. After 24 h, cells were treated with various concentrations of the tested compounds in triplicates. After 24 h, the supernatant was discarded, and cell monolayers were washed with sterile 1x phosphate buffer saline (PBS) 3 times, and MTT solution (20 μL of 5 mg/ml stock solution) was added to each well and incubated at 37 °C for 4 h followed by medium aspiration. In each well, the formed formazan crystals were dissolved with 200 μL of acidified isopropanol

(0.04 M HCl in absolute isopropanol = 0.073 ml HCL in 50 ml isopropanol). The absorbance of formazan solutions was measured at λmax 540 nm with 620 nm as a reference wavelength using a multi-well plate reader. The percentage of cytotoxicity compared to the untreated cells was determined with the following equation.

The plot of % cytotoxicity versus sample concentration was used to calculate the concentration which exhibited 50% cytotoxicity (TC_{50}) [1].

$$\%\ \text{cytotoxicity} = \frac{(\text{The absorbance of cells without treatment} - \text{absorbance of cells with treatment})\ \text{X}\ 100}{\text{The absorbance of cells without treatment}} \quad (1)$$

2.3. Inhibitory Concentration 50 (IC_{50}) Determination

In 96-well tissue culture plates, 2.4 × 104 Vero-E6 cells were distributed in each well and incubated overnight in a humidified 37 °C incubator under 5% CO_2 condition. The cell monolayers were then washed once with 1x PBS and subjected to virus adsorption for 1 h at RT. The cell monolayers were further overlaid with 50 µl of DMEM containing varying concentrations of the tested compounds. Following incubation at 37 °C in a 5% CO_2 incubator for 72 h, the cells were fixed with 100 µl of 4% paraformaldehyde for 20 min and stained with 0.1% crystal violet in distilled water for 15 min at RT. The crystal violet dye was then dissolved using 100 µl absolute methanol per well and the optical density of the color measured at 570 nm using Anthos Zenyth 200rt plate reader (Anthos Labtec Instruments, Heerhugowaard, Netherlands). The IC_{50} of the compound is that required to reduce the virus-induced cytopathic effect (CPE) by 50%, relative to the virus control.

3. Results

Table S1. The scores of re-docking the co-crystallized ligands and the top-scoring compounds of the strawberry and ginger methanolic extracts against seven SARS-CoV-2 protein targets.

**Compound/Target**	**HUMAN**			**VIRAL**			
	**AAK1 (5L4Q) Kcal/mol**	**CATHEPSIN L (5MQY) Kcal/mol**	**Mpro (6LU7) Kcal/mol**	**ADP Ribose Phosphatase (6W02) Kcal/mol**	**NSP 14 (5C8S) Kcal/mol**	**NSP 16 (6W61) Kcal/mol**	**PLpro (4OW0) Kcal/mol**
**Co-crystallized ligands**	−10.2	−8.7	−13.6	−10.1	−11.5	−7.8	−10
**Neohesperidin**	−10.4	−7.3	−8.8	−9.6	−9.6	−9.3	−7.2
**Isovitexin-2''-*O*-rhamnoside**	−9.4	−7.9	−8.3	−8.5	−8.8	−9.2	−7.3
**Epicatechin 5-*O*-β-D-glucopyranoside-3-benzoate**	−8.3	−7.7	−9.7	−9.7	−9.7	−7.3	−8.3
**Quercetin 3-*O*-neohesperidoside**	−8.9	−7.1	−8.6	−9.4	−10.3	−7.9	−7.8
**Kaempferitrin**	−9	−7.9	−8.8	−7.5	−9.6	−9.2	−8.9
**β- amyrin**	−8.3	−7.4	−7.3	−8	−9.1	−8.3	−6
**Apigenin**	−8.9	−6.4	−7.8	−8.7	−8.6	−7.6	−7.6
**Epicatechin**	−8.1	−5.7	−7.2	−8.3	−8.9	−8.1	−7.2
**Quercetin**	−9	−6.7	−7.7	−8.4	−8.9	−8.2	−7.7
**5,6-epoxy cholestan-3-ol**	−8.4	−6.5	−6.8	−8	−9.2	−7.5	−7.3
**Riboflavin**	−8.8	−6.8	−8	−7.4	−9.5	−7	−7.8
**1,7-bis-(4-Hydroxy-3-methoxyphenyl)-hepta-1,6-diene-3,5-dione**	−7.9	−6.9	−7.3	−8.4	−9.2	−6.8	−7.9
**Gingerenone-A**	−8.2	−6.3	−7	−8.6	−8.7	−6.5	−7.6

References

1. Sarkar, S. Silver Nanoparticles with Bronchodilators Through Nebulisation to Treat COVID 19 Patients. *J. Curr. Med Res. Opin.* **2020,** *3*, 449–450.
